# Synthesis of di- and tri-cellulose acetate from rice husk cellulose and commercial microcrystalline by copper perchlorate catalyst

**DOI:** 10.1038/s41598-026-53816-6

**Published:** 2026-05-27

**Authors:** Safaa Ragab, Amany El Sikaily, Ahmed El Nemr

**Affiliations:** National Institute of Oceanography and Fisheries (NIOF), Kayet Bey, Elanfoushy, Alexandria Egypt

**Keywords:** Copper(II) perchlorate hexahydrate, Microcrystalline cellulose, Cellulose acetate, Rice husk cellulose, Acetylation, Chemistry, Materials science

## Abstract

This work describes the synthesis of various di- and tri-cellulose acetates from rice husk cellulose (RHC) and commercial microcrystalline cellulose (CMCC) using copper(II) perchlorate hexahydrate (Cu(ClO_4_)_2_·6H_2_O) as an effective catalyst at room temperature and 50 °C. This investigation was conducted using different amounts of Cu(ClO_4_)_2_·6H_2_O (100, 200, 300 mg) and 2 g of cellulose for various times (0.5–6 h) in the presence of a constant volume of acetic anhydride (15 mL). Multiple reactions have led to the formation of di- and tri-cellulose acetates. CMCC was remarkably converted into cellulose acetate at room temperature and 50 °C, with yields of 98.10% and 96.10%, respectively. The extracted cellulose from rice husk produced di- and tri-cellulose acetate at room temperature and 50 °C, yielding 86.93% and 92.85%, respectively. Critical expected results were obtained in this work: a strong relationship was found between the degree of substitution (DS) and acetyl percentage (AP%) of the products, the catalyst level in the reaction mixture, the presence or absence of temperature, and the reaction time. The DS and AP% were characterized using FTIR, ^1^H-NMR, XRD, and the thermal stability was evaluated by TGA and DTA. This work presents a new catalyst that can produce varying degrees of cellulose acetylation by adjusting reaction temperature, catalyst amount, and reaction duration.

**Supplementary Information:**

The online version contains supplementary material available at 10.1038/s41598-026-53816-6.

## Introduction

Increasing concern about the environment and the partial replacement of synthetic materials with biodegradable ones has heightened interest in cellulose manufacturing^1^. Investigating new sources and more sustainable, efficient cellulose extraction methods is imperative, given current economic challenges and the rising demand for cellulosic products. Water treatment, biomaterial composites, textile and paper production, food packaging, and the pharmaceutical sector are just a few industries that use cellulose extensively as a raw material^2^.

When rice is ground, one of the main agricultural residues left over is rice husk (RH). According to the Food and Agriculture Organization of the United Nations (FAO), RH accounted for almost 23% of the 466 million tons of rice produced worldwide in 2010–2011^3^. The lignocellulosic material known as rice husk, which is made from agricultural waste, is one of the biomass materials with the highest chemical content of organic carbon (45–50%)^4^. Using rice husk as the primary source for cellulose fiber production is promising, as it contains 33% cellulose, 26% hemicellulose, and 7% lignin^5^. Several attempts to extract cellulose from rice husk have been performed using classical chemical treatments, including alkali and bleaching treatments^6^. Additionally, silica and cellulose can also be extracted from rice husk^7^.

Because cellulose is abundant in nature, biodegradable, and has a lower environmental impact than polymers derived from fossil fuels, its manufacture has drawn much attention recently^8^. Waste management, oil extraction, paper production, textile finishing, culinary additives, and medicinal applications all use cellulose derivatives extensively^9^. Cellulose acetate is a key derivative of cellulose, the most industrially important, with approximately 1.5 billion pounds manufactured each year globally^10^.

Due to the effectiveness of heterogeneous catalysts from both economic and ecological standpoints, they have gained significance in the acetylation of cellulose under solvent-free conditions in recent years^11^.

Among the metal perchlorates, LiClO_4_^12^, Mg(ClO_4_)_2_^13^, BiOClO_4_^14^, and Zn(ClO_4_)_2_·6H_2_O^15^ were used for the protection of heteroatoms. Additionally, LiClO_4_ was utilized as a catalyst for the acetylation of alcohols and phenols with acetic anhydride, yielding excellent results, as well as for the acylation of various activated benzenes^16,17^. Zn(II) perchlorate hexahydrate was used as a powerful catalyst for the conjugate addition of thiols to U, P-unsaturated ketones at room temperature, without the need for a solvent, and for the acylation of alcohols in the presence of acid anhydrides^18^.

Copper(II) perchlorate is an inorganic chemical compound formed from a salt of copper and an acid reaction, which can be used as a catalytic oxidant to form a copper complex^19^. More recently, we proposed ferric perchlorate hydrate as a valuable catalyst for the acetylation process of microcrystalline cellulose with varying degrees of substitution under simple conditions^20^. These promising results motivated us to explore the use of other metal perchlorates in the acylation process of various types of cellulose. To our knowledge, no report exists that utilizes perchlorate to protect aldehydes. Moreover, transition-metal perchlorates are not used for the acylation of heteroatoms. We employed copper(II) perchlorate hexahydrate as an efficient catalyst in this manner, which proved more practical from both economic and ecological standpoints. Copper(II) perchlorate hexahydrate offers lower toxicity (Cu^2+^ is an essential micronutrient at trace levels) and aligns with green chemistry principles. These benefits are particularly relevant for scalable water treatment applications. This article describes a more effective and environmentally friendly method of producing di- and tri-cellulose acetate by reacting easily accessible and reasonably priced starting materials, such as commercial microcrystalline cellulose (CMCC) and extracted rice husk cellulose (RHC), with a small amount of copper perchlorate hexahydrate acting as a catalyst in a solvent-free environment. The main approaches to acetylating cellulose include integrating an effective catalyst, producing various types of di- and tri-acetyl cellulose, and minimizing the amount of acetic anhydride (15 mL) at both room temperature and 50 °C.

## Materials and methods

### Materials and chemicals

The local market supplied the dry rice husk powder used in the reactions (Alexandria, Egypt). All reagents and chemicals used in this work were of standard analytical grade: acetic anhydride, sodium hypochlorite, copper(II) perchlorate hydrate, hydrogen peroxide, ethanol, and sodium hydroxide, purchased from Merck.

### Extraction of cellulose from rice husk powder

Two steps of delignification were used to extract the cellulose from rice husk. The first step was alkali treatment, which involved heating a dry rice husk sample in a water bath for two hours at 70 °C while treating it with a sodium hydroxide solution (5% weight) (solid-liquid ratio: 1:10 (W/V)). After the mixture cooled, the biomass was separated by filtration and repeatedly washed with distilled water. The second step involved bleaching with a 2% sodium hypochlorite (NaOCl) solution at a 1:10 (w/v) ratio and heating it in a water bath for 2 h at 70 °C. The bleaching mixture was then filtered and repeatedly cleaned with distilled water. The cellulose component was purified by two iterations of the NaOCl bleaching procedure. The bleaching procedure was carried out at 70 °C using 5% H_2_O_2_ at a 1:10 (W/V) ratio. Following the bleaching process, the biomass was repeatedly cleaned with distilled water. The product was oven-dried at 50 °C for 24 h after filtering. Rice milling yields ~ 36% microcrystalline cellulose (MCC) from rice husks. Rice husk MCC generally has particle sizes between 75 and 240 μm, with processing-induced flake-like or uneven forms and surface areas. Rice husk MCC generally has particle sizes between 75 and 240 μm, with processing-induced flake-like or uneven forms and surface areas. Rice-derived MCC typically has larger particles but similar flowability and compressibility to commercial MCC (mean ~ 50 μm, rod-spherical form, ~ 0.3–0.4 g/cc bulk density).

The extraction process, including alkali treatment, bleaching conditions, washing, and drying, was thoroughly explained to ensure the reliability and reproducibility of the extracted rice husk cellulose. FTIR, XRD, and extraction yield measurements were used to assess the successful isolation. While the absence of absorption from non-cellulosic components demonstrated the successful removal of hemicellulose/lignin-derived contaminants, the extracted cellulose’s FTIR spectrum showed the characteristic cellulose bands. The cellulose structure was further validated by XRD analysis, which revealed that the isolated rice husk cellulose had a crystallinity index of 30.2%. These results confirm that the extracted cellulose is a suitable precursor for the subsequent acetylation processes.

### Synthesis of di- and tri-cellulose acetate from commercial microcrystalline and extracted rice husk cellulose

The synthesis of the cellulose acetates (CA) from commercial microcrystalline and extracted rice husk cellulose (RHC) was carried out according to the free solvent method at room temperature by the addition of 100, 200, and 300 mg of the catalyst of Cu(ClO_4_)_2_.6H_2_O and 15 mL of acetic anhydride to 2.0 g of the microcrystalline and rice husk cellulose. The reaction mixture was stirred at room temperature for 1, 2, 3, 4, and 6 h. After that, 30 mL of ethanol was slowly added to the reaction medium by stirring it with a glass rod. The solution was left for 30 min, and 100 mL of distilled water was added to the mixture until a precipitate formed directly. The di- and tri-cellulose acetate products were obtained, and then the precipitate was vacuum-filtered and washed with distilled water and ethanol. Finally, the product was oven-dried overnight at 50 °C. All di- and tri-cellulose acetate production was performed at room temperature^20^. In brief, the acetylation of microcrystalline cellulose and rice husk cellulose was conducted similarly, but at 50 °C rather than room temperature, for 0.5, 1, 2, and 3 h using the aforementioned process. The recyclability of Cu(ClO_4_)_2_·6H_2_O was not examined in this paper, which is now recognized as a study limitation. Since the catalyst is soluble in the reaction media and mostly stays in the liquid phase following cellulose acetate precipitation, further separation, purification, and quantitative investigation of catalyst retention or copper leaching would be necessary for its recovery. Therefore, future research should concentrate on catalyst recovery and reuse, including assessing catalytic activity throughout subsequent cycles and how it affects yield, DS, AP%, and product purity.

A progressive increase in temperature was not used for the acetylation processes. To assess the impact of temperature under controlled conditions, two fixed temperatures (room temperature and 50 °C) were selected. The effects of catalyst quantity and reaction duration were methodically examined at each temperature. Reactions for CMCC were carried out using 100, 200, and 300 mg of Cu(ClO_4_)_2_·6H_2_O at room temperature for 1–4 h and at 50 °C for 0.5–3 h. The same catalyst quantities were used for RHC reactions, which were carried out at room temperature for 1–6 h and at 50 °C for 0.5–3 h. As indicated in Tables 1, 2, 3 and 4, the optimization was based on product yield, acetyl percentage, DS calculated by FTIR, and DS estimated by titration.

### Determination of degree of substitution (DS) and Acetyl percentage % (AP)

The degree of substitution (DS) was the average value of (CH_3_COO) groups in every glucose unit in the cellulose chain. It can be determined by FT-IR spectroscopy and also by titration with aqueous NaOH solution^21^. According to Puleo et al. (1989)^22^, the experimental degree of substitution (DSt), it is possible to calculate the acetyl percentage (AP%) because a degree of substitution of 2.88 corresponds to an AP% of 43.5%^21,22^.

Using the established relationship between DS and acetyl content in cellulose acetate, the acetyl % was computed using the experimentally obtained degree of substitution. Increased DS values are closely correlated with increased acetyl content because DS represents the average number of substituted hydroxyl groups per anhydroglucose unit. As a result, an AP% of roughly 43.5% corresponds to a DS value of roughly 2.88, which is consistent with substantially substituted cellulose acetate. The close agreement between DS values obtained by titration and FTIR, as well as the ^1^H-NMR integration data, which verified the existence of anhydroglucose ring protons at 3.5–5.1 ppm and acetyl methyl protons at 1.9–2.1 ppm, provided support for this assignment.

### Characterization of prepared di- and tri-cellulose acetate

A Bruker model Vertex 70 spectrometer, connected to a platinum ATR unit (Bruker, Germany), was used to analyze all samples using FT-IR spectroscopy in the 4000 –400 cm^–1^ spectral range. ^1^H NMR in CDCl_3_ was recorded on a JEOL NMR spectrometer at 500 MHz. Thermal experiments were conducted between 50 and 900 °C at a flow rate of 100 mL/min of N2. Thermogravimetric analysis was performed using a TA Instruments SDT650 at a heating rate of 10 °C/min from 30 to 1000 °C under a nitrogen atmosphere. A Panalytica X-Ray Diffractometer with Cu Kα radiation (k = 0.15406 nm) and a scanning range of 2̟ (0–90) was used to perform the powder X-ray (XRD) measurement. At room temperature, tetrahydrofuran was used as the mobile phase at a flow rate of 1.0 mL/min in gel permeation chromatography (GPC) (Agilent Technologies-1260 Infinite II series) to determine the molecular weight, degree of polymerization, and polydispersity of a substance. At room temperature, tetrahydrofuran was used at a flow rate of 1.0 mL/min as the mobile phase in gel permeation chromatography (GPC) (Agilent Technologies-1260 Infinite II series) to determine the molecular weight, degree of polymerization, and polydispersity of a substance (Table S1)^23^.

## Results and discussion

### Optimization of the reaction conditions

The primary objective of this study was to develop a novel acetylation procedure utilizing a highly reactive catalyst, specifically Cu(ClO_4_)_2_**·**6H_2_O. Another important goal was to test the success of this catalyst by applying it to two types of cellulose: commercial microcrystalline cellulose and extracted RHC, at room temperatures (20 ± 2 °C) and 50 °C. To assess the efficiency and appropriateness of previously reported procedures for the acetylation of cellulose with metal perchlorate hydrate, we selected the reaction variables to study, including reaction temperature, reaction time, and catalyst level^20^.

#### Temperature

The effect of temperature on the reactions was investigated by conducting them at room temperature and 50 °C. The yield % of di- and tri-cellulose acetate obtained from commercial microcrystalline and extracted RHC increased significantly with increasing reaction temperature from room temperature to 50 °C. The optimum conditions for the acetylation reaction of CMCC at room temperature were 4 h and 300 mg of catalyst. At 50 °C, the optimum conditions were 2 h and 100 mg of catalyst. Therefore, sample 12 was considered optimal (yield% = 98.10% and DS = 2.94) at room temperature, and sample 15 was considered optimal at 50 °C (yield% = 96.10% and DS = 2.88), respectively. In addition, the optimum conditions for acetylation reaction of extracted RHC at room temperature were 6 h and level of catalyst was 300 mg while when the temperature rises to 50 °C, the optimum conditions (time = 3 h and the level of catalyst = 300 mg). Therefore the sample 39 was considered as optimal at room temperature (the yield % = 86.93 and DS = 2.61). The sample 51 was counted as optimal at 50 °C (the yield % = 92.85 and DS = 2.79), respectively. The results from Tables (1–4) indicate the critical role the temperature plays. For example, in the acetylation of CMCC, the yield % increases (Sample 1) from 2.50 g at room temperature to 3.35 g (Sample 14) at 50 °C, while the rest of the reaction conditions remain the same (Tables 1 and 2). The yield % and DS increased with increasing temperature, but due to the catalyst’s high reactivity, a lower reaction temperature (50 °C) was used. Importantly, in the acetylation reaction of extracted RHC at room temperature, the di-cellulose acetate only formed, even when the time of reaction was extended to 6 h (Samples 25–39, Table 3), while the rising temperature to 50 °C improved the yield % and the tri-cellulose acetate was obtained (Tables 3 and 4). It was proposed that increasing the temperature up to 50 °C promoted the acetylation of the surface of commercial microcrystalline and extracted RHC by accelerating the reaction rate, dissolving the formed cellulose acetate, and allowing the remaining unreacted hydroxyl groups to react. The DSt, DSFT-IR, AP%, and yield% were calculated for each product, and the data are reported in Tables 1, 2, 3 and 4.

**Table 1:** Acetylation of 2.0 g of microcrystalline cellulose in the presence of different weights of catalyst Cu(ClO_4_)_2_.6H_2_O at room temperature and in a constant volume of acetic anhydride (15 mL).

Sample No.	Cu(ClO_4_)_2_.6H_2_O (mg)	Time (h)	Yield (g)	% yield	Degree of acetylation (DS)_t_	Degree of acetylation (DS)_FT−IR_	Acetyl percentage % (AP)
1	100	1	2.50	70.13	2.10	2.15	31.72
2	100	2	2.65	74.41	2.23	2.45	38.68
3	100	3	2.90	81.23	2.44	2.32	36.85
4	100	4	3.05	85.86	2.58	2.57	38.97
5	200	1	2.74	76.90	2.31	2.35	34.89
6	200	2	2.99	83.81	2.51	2.44	37.91
7	200	3	3.19	89.81	2.69	2.61	40.63
8	200	4	3.30	92.68	2.78	2.78	41.99
9	300	1	2.78	80.05	2.34	2.33	35.34
10	300	2	3.05	85.70	2.57	2.55	38.82
11	300	3	3.27	91.80	2.75	2.74	41.54
12	300	4	3.49	98.10	2.94	2.93	44.41

(DS)_t_: Degree of acetylation by titration; (DS)_FT−IR_: Degree of acetylation by FT-IR.

**Table 2:** Acetylation of 2.0 g of microcrystalline cellulose in the presence of different weights of the catalyst Cu(ClO_4_)_2_.6H_2_O at 50 °C and in a constant volume of acetic anhydride (15 mL).

Sample No.	Cu(ClO_4_)_2_.6H_2_O (mg)	Time (h)	Yield (g)	% yield	Degree of acetylation (DS)_t_	Degree of acetylation (DS)_FT−IR_	Acetyl percentage % (AP)
13	100	0.5	2.99	84.16	2.52	2.52	38.06
14	100	1	3.35	94.11	2.82	2.80	42.59
15	100	2	3.42	96.10	2.88	2.87	43.5
16	100	3	3.40	95.53	2.78	2.75	41.99
17	200	0.5	3.32	93.30	2.80	2.79	42.30
18	200	1	3.40	95.44	2.86	2.84	43.20
19	200	2	3.41	95.96	2.88	2.84	43.5
20	200	3	3.28	92.25	2.77	2.75	41.83
21	300	0.5	3.33	93.72	2.82	2.82	42.59
22	300	1	3.37	94.84	2.85	2.86	43.05
23	300	2	3.32	93.46	2.80	2.82	42.29
24	300	3	3.24	90.98	2.73	2.77	41.23

**Table 3:** Acetylation of 2.0 g of extracted RHC in the presence of different weights of the catalyst Cu(ClO_4_)_2_.6H_2_O at room temperature and in a constant volume of acetic anhydride (15 mL).

Sample No.	Cu(ClO_4_)_2_.6H_2_O (mg)	Time (h)	Yield (g)	% yield	Degree of acetylation (DS)_t_	Degree of acetylation (DS)_FT−IR_	Acetyl percentage % (AP)
25	100	1	2.07	58.33	1.75	1.70	26.43
26	100	2	2.18	61.22	1.84	1.81	27.79
27	100	3	2.23	62.72	1.88	1.85	28.40
28	100	4	2.30	64.63	1.94	1.93	29.30
29	100	6	2.87	80.77	2.42	2.40	36.55
30	200	1	2.23	62.70	1.88	1.85	27.94
31	200	2	2.32	65.31	1.96	1.94	29.30
32	200	3	2.56	71.91	2.16	2.11	31.87
33	200	4	2.72	76.58	2.30	2.31	34.89
34	200	6	2.87	80.57	2.42	2.40	36.25
35	300	1	2.36	66.33	1.99	1.99	30.06
36	300	2	2.50	70.42	2.11	2.10	31.72
37	300	3	2.87	80.78	2.42	2.40	36.25
38	300	4	3.04	85.37	2.56	2.54	38.36
39	300	6	3.09	86.93	2.61	2.60	39.27

**Table 4:** Acetylation of 2.0 g of RHC in the presence of different weights of the catalyst Cu(ClO_4_)_2_.6H_2_O at 50 °C and in a constant volume of acetic anhydride (15 mL).

Sample No.	Cu(ClO_4_)_2_.6H_2_O (mg)	Time (h	Yield (g)	% yield	Degree of acetylation (DS)_t_	Degree of acetylation (DS)_FT−IR_	Acetyl percentage % (AP)
40	100	0.5	2.28	63.96	1.92	1.90	29.00
41	100	1	2.35	66.16	1.95	1.93	29.45
42	100	2	2.46	69.11	2.10	2.10	31.72
43	100	3	2.53	71.11	2.13	2.10	32.17
44	200	0.5	2.13	59.90	1.80	1.80	27.19
45	200	1	2.47	69.24	2.11	2.11	31.87
46	200	2	3.02	84.91	2.55	2.54	38.52
47	200	3	3.17	89.23	2.68	2.68	40.48
48	300	0.5	2.69	75.63	2.27	2.27	34.29
49	300	1	2.89	81.28	2.44	2.42	36.85
50	300	2	3.30	92.72	2.79	2.78	42.14
51	300	3	3.30	92.85	2.79	2.78	42.14

#### Time

To determine the optimal conditions for the acetylation of commercial microcrystalline and extracted RHC, the reactions were conducted over different reaction times (0.5–4 h and 0.5–6 h, respectively). Remarkably, the yield % and DS for acetylation of CMCC at room temperature increased with the extended reaction time from 1 to 4 h. As we explained previously, the reaction condition used to obtain sample 12 was deemed optimal for the acetylation of CMCC at room temperature. In contrast of, in acetylation of CMCC at 50 °C, the yield (%) and DS increased as the time extended from 0.5 to 2 h, but after that, when the time of reaction extent to 3 h, the yield (%) beginning to decreased and the optimum conditions for acetylation reaction of CMCC at 50 °C were 2 h and 100 mg of catalyst, and the reaction condition used for sample 15 was considered as optimal reaction condition.

By comparing the yield of acetylation reaction of CMCC, Sample 11 (T = room temperature, time = 3 h and Wt. of catalyst = 300 mg, yield (3.27 g)) and sample 24 (T = 50 °C, time = 3 h and Wt. of catalyst = 300 mg, yield (3.24 g)) we can evidently deduced that, the extended of the time reaction at 50 °C from 2 to 3 h leads to the hydrolysis of the formed cellulose acetate, Tables (1 and 2). Regarding the yield % and DS for the acetylation of extracted RHC, whether at room temperature or 50 °C, the yield increased with extended reaction time from 0.5 to 6 h. The optimal conditions for the acetylation reaction of extracted RHC at room temperature were 6 h and 300 mg of catalyst. Sample 39 was considered optimal. At 50 °C, Sample 51 was considered optimal, as explained before (Tables 3 and 4).

#### Catalyst (copper (II) perchlorate hydrate)

The reactions were performed with three different levels of Cu(ClO_4_)_2_·6H_2_O (100, 200, and 300 mg) to illustrate the effect of catalyst loading on acetylation. The effects of catalyst level on yield % and degree of substitution (DS) of acetate for different types of cellulose were summarized in Tables 1, 2, 3 and 4. The results of all the Samples (1–12) for the acetylation of commercial microcrystalline at room temperature and extracted RHC at room temperature and 50 °C (25–51) have shown that, the yield % and DS of the di- and tri-cellulose acetate were increased with the increasing the amount of copper (II) perchlorate hydrate from 100 to 300 mg (Tables 1, 3 and 4). Except for samples of the acetylation of CMCC at 50 °C (Samples 13–24), the yield (%) and the degree of substitution (DS), an excess of the amount of catalyst from 100 to 300 mg led to a decrease in the yield (Table 2). From the results in Table 2, we observed that 100 mg of the catalyst was sufficient to complete the reaction within 2 h at 50 °C with commercial microcrystalline (Sample 15). It is well known that an excess of acetic anhydride, with increasing the level of catalyst, leads to the formation of more intermediates, and cellulose is more easily acetylated^20,23–27^. However, in this context, the amount of AC_2_O reagent was fixed under all acetylation reactions. Notably, the reactions proceed even with that amount of the AC_2_O reagent (15 mL). The data in Tables 1, 2, 3 and 4 demonstrate that our procedure is convenient and effective for the acetylation of commercial microcrystalline and extracted RHC at both room temperature and 50 °C. In fact, whereas under solvent-free conditions di- and tri-cellulose acetate can be easily formed, it was proposed that, the catalyst Cu(ClO_4_)_2_·6H_2_O, which is very reactive serves to create a very fast acetic anhydride-Cu(ClO_4_)_2_·6H_2_O intermediate at the beginning of the reaction through the activation of the C = O of acetic anhydride, then the oxygen atom of the cellulose can easily attack the carbonyl carbon of acetic anhydride via its lone pairs, and the acetic acid molecule is removed, allowing the acetylation process to continue^20^. Remarkably, this expedient avoids the use of a large amount of catalyst, reagent, and high reaction temperature.

### Characterization

#### FTIR

**Fig. 1:**
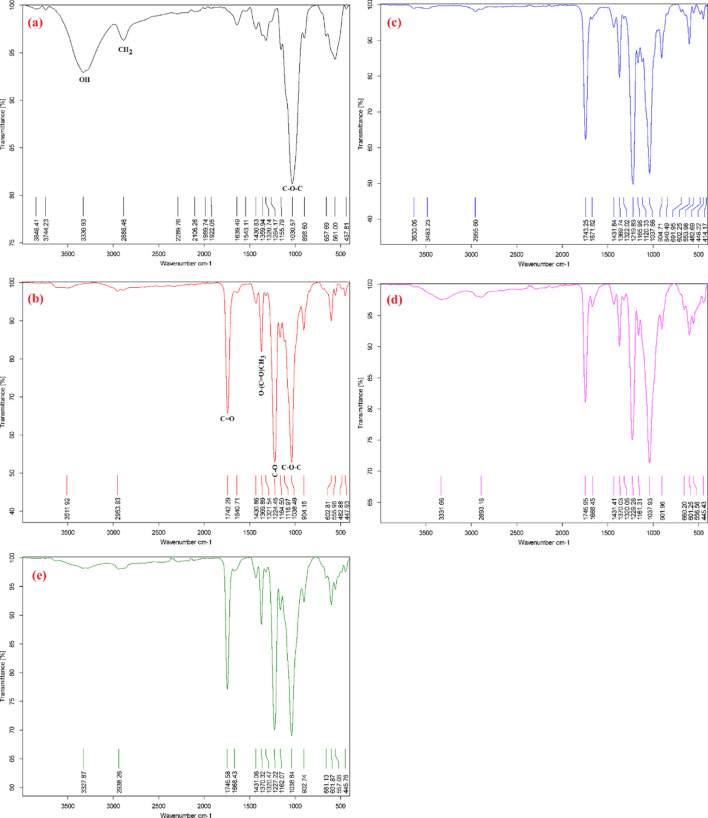
FTIR analysis of (**a**) rice husk microcrystalline cellulose, (**b**) reaction 12, (**c**) reaction 15, (**d**) reaction 39, and (**e**) reaction 51.

FTIR analysis of extracted RHC and synthesized cellulose acetate of commercial microcrystalline and extracted rice husk at room temperature and 50 °C was performed to observe the appearance of ester bands, hydroxyl group absorptions, and for comparison between the non-modified cellulose and acetylated modified cellulose. The obtained spectra are presented in Fig. 1(a-e). The absorbance bands at 3331, 2888, 1640, 1360, 1156, 1031, and 658 cm^− 1^ in Fig. 1(a) are associated with a non-modified extracted RHC molecule. The band at 3331 cm^− 1^ is assigned to free O-H stretching vibration groups. The band at 2888 cm^− 1^ corresponds to C-H stretching groups, while the band at 1640 cm^–1^ is probably associated with the bending mode of the adsorbed water in the non-modified extracted RHC molecule. The band at 1360 cm^–1^ is due to O-H bending, and the band at 1156 cm^− 1^ is related to the C-O anti-symmetric bridge stretching groups. The absence of a band around 1210 cm^–1^ indicated the elimination of non-cellulosic polysaccharide groups^24^. In comparison, the three crucial characteristic ester bands were easily recognized in the modified commercial microcrystalline FT-IR spectra and extracted RHC at room temperature and 50 °C (Fig. 1b-e). The band at (1739–1746 cm^–1^) is attributed to the carbonyl C = O stretching vibration groups, the carbon-hydrogen bond in the methyl group in plane bending in O-(C = O)-CH_3_ at (1369–1370 cm^− 1^), and the carbon oxygen bond stretching vibration of the acetyl group at (1218–1220 cm^–1^). A strong band at (1037–1038 cm^− 1^) is due to the C-O-C pyranose ring’s skeletal vibration. In addition, the disappearance of the band at 3331 cm^− 1^, which is assigned to free O-H stretching vibration groups, and the appearance of the three characteristic ester bands in spectra in (Fig. 1b-e) provide strong evidence for the successful acetylation reaction. Additionally, the disappearance of the absorption band at 1700 cm^− 1^, corresponding to the carboxylic group, indicates that the product is released from unreacted acetic anhydride or acetic acid byproduct^25,26^.

#### ^1^H-NMR

**Fig. 2:**
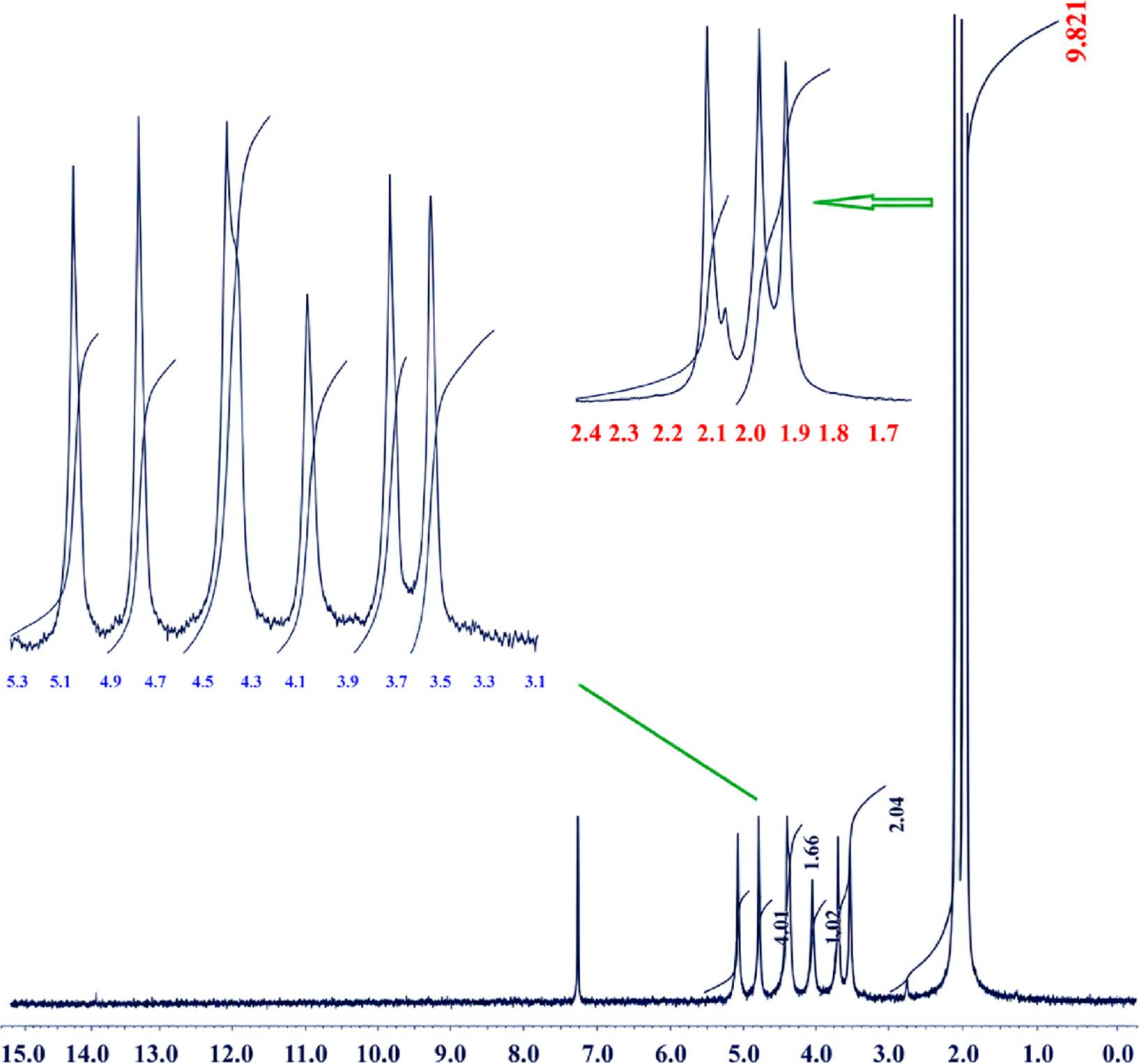
^1^H-NMR analysis of Sample 12.

The ^1^H NMR spectrum was used to confirm the successful acetylation of commercial microcrystalline cellulose and to determine the degree of substitution (DS) of the produced cellulose acetate. Figure 2 presents the ^1^H NMR spectrum of Sample 12, which was synthesized under the optimized conditions of room temperature, 4 h reaction time, and 300 mg of Cu(ClO_4_)_2_·6H_2_O catalyst, giving a high product yield of 98.10%. As shown in Fig. 2, the spectrum displays the characteristic proton resonances of cellulose acetate. The intense signals observed in the region of approximately 1.9–2.1 ppm are assigned to the methyl protons of the acetyl groups (–OCOCH_3_). The enlarged inset of this region clearly shows the presence of multiple acetyl methyl proton signals, indicating that acetyl groups are distributed at different hydroxyl positions of the anhydroglucose unit. In cellulose acetate, these methyl signals are commonly associated with acetyl substitution at the C2, C3, and C6 positions. Their strong intensity confirms the extensive introduction of acetyl groups onto the cellulose backbone. The signals appearing in the region of approximately 3.5–5.1 ppm are attributed to the ring protons of the anhydroglucose unit. The expanded part of the spectrum in this region shows several well-defined resonances, which correspond to the different protons on the cellulose backbone after acetylation. The downfield signals near 4.7–5.1 ppm can be related to ring protons affected by neighboring acetyl substituents, while the signals between 3.5 and 4.5 ppm are assigned to the remaining anhydroglucose ring protons. The appearance of these signals, together with the acetyl methyl resonances, confirms the formation of cellulose acetate. The degree of substitution was calculated from the integration ratio between the acetyl methyl proton region and the anhydroglucose ring proton region. In this calculation, one-third of the integrated area of the acetyl methyl proton signals at 1.9–2.1 ppm was divided by one-seventh of the integrated area of the ring proton signals at 3.5–5.1 ppm, according to the number of protons contributing to each region. Based on the integration values shown in Fig. 2, the DS value was calculated to be 2.94. This value is very close to the theoretical maximum DS of 3.0 for cellulose triacetate, indicating that most of the available hydroxyl groups in the cellulose structure were successfully converted into acetyl ester groups. Therefore, the ^1^H NMR results confirm that the Cu(ClO_4_)_2_·6H_2_O-catalyzed acetylation system is highly efficient under mild room-temperature conditions. The high DS value of 2.94, together with the high yield of 98.10%, demonstrates the successful preparation of highly substituted cellulose acetate from commercial microcrystalline cellulose^27,28^.

#### XRD

**Fig. 3:**
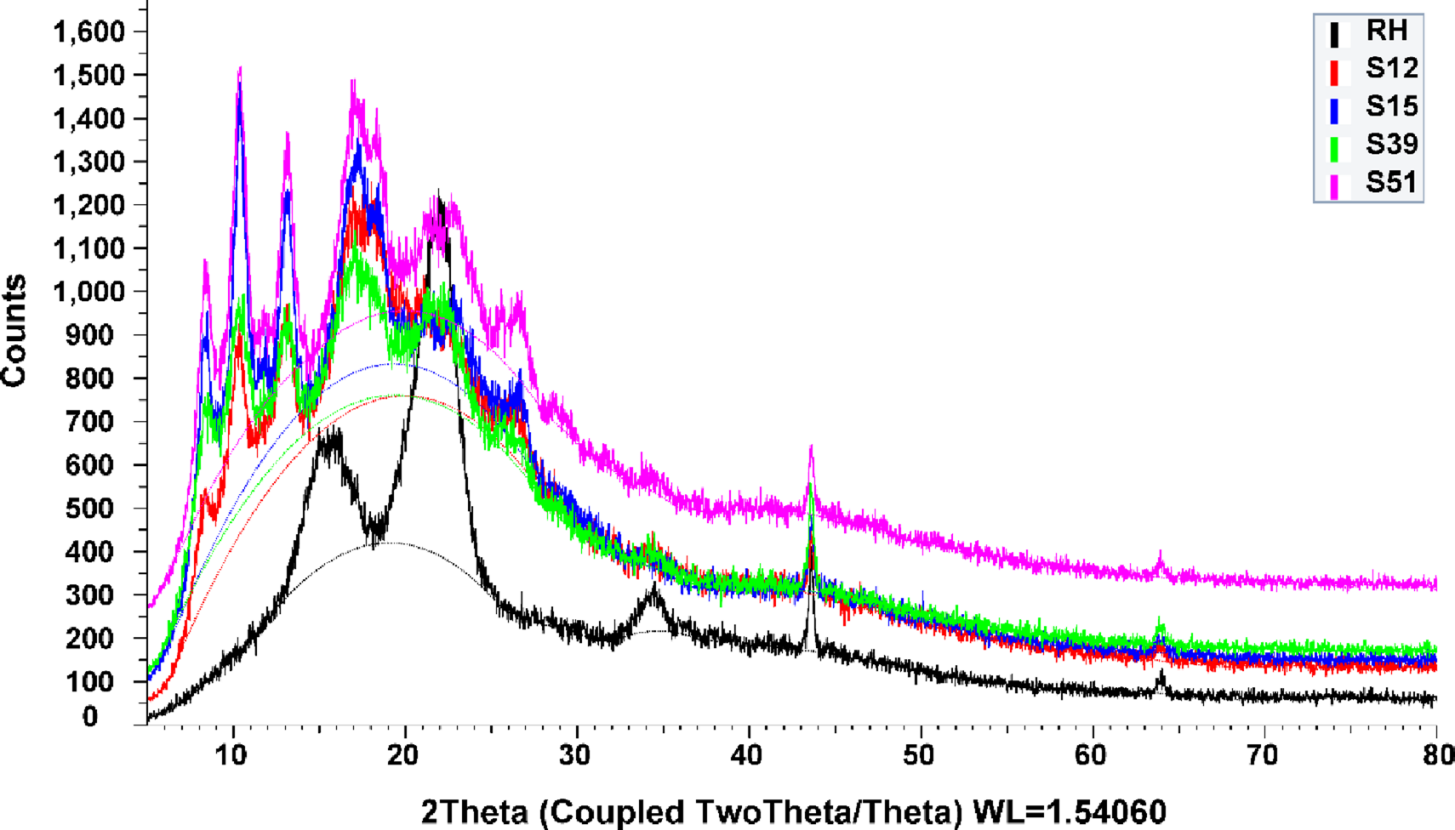
The XRD profile of extracted RHC and samples 12, 15, 39 and 51.

XRD analysis was used to confirm the effect of acetylation on the crystallinity and structure of the extracted rice husk and microcrystalline cellulose. The XRD profiles comparison of extracted RHC and synthesized cellulose acetate of commercial microcrystalline and extracted rice husk at room temperature and 50 °C (samples 12, 15, 39, and 51, respectively) are presented in Fig. 3. The XRD profile of extracted RHC shows peak diffraction localized at 2*θ* = 16^o^, and 34^o^, these peaks are assigned to cellulose ICDD # 00-003-0226. The identified silica phase, assigned to ICDD # 00-001-0424, was localized around 2*Ɵ* = 22°. On comparing the results of cellulose acetate obtained from acetylation of commercial microcrystalline and extracted rice husk at room temperature and 50 °C (samples 12, 15, 39 and 51, respectively), the XRD pattern indicated similar diffraction peaks. In Fig. 3, samples 12, 15, 39,, and 51 showed less intense peaks at 8.4°, 10.6°, 17.4°, and 20.0°, respectively. The peak diffraction localized at 8.4° is assigned to semicrystalline acetylated cellulose. The diffraction peak around 10.6° is associated with the crystalline peaks of cellulose acetate II^29^. The diffraction peak around 20° was usually observed in all organic polymers and was assigned to the Van der Waals or amorphous halo (amorphous region) of the cellulose chains^30^. Cellulose acetate (samples 12, 15, 39, and 51, respectively) presented a lower degree of crystallinity (21.5, 23.6, 22.7, and 24.8%, respectively), as compared with extracted RHC (30.2%). This reduction in crystallinity takes place due to the replacing of the hydroxyl groups by acetyl groups along the axes of cellulose chain, it’s known, the acetyl group has a greater volume, which causing an increase in the interfibrillar distance, breakdown of microfibrilllar structures, breakdown of the inter- and intra-molecular hydrogen bonds of the cellulose resulting in a disorder in the cellulose structure^31,32^. The crystallinity of the extracted RHC (crystallinity = 30.2%) was significantly higher than that of the synthesized cellulose acetate of commercial microcrystalline and extracted rice husk at room temperature and 50 °C (crystallinity of samples 12 (21.5%), 15 (23.6%), 39 (22.7%), and 51 (24.8%)).

#### Thermo gravimetric analysis (TGA) and derivative thermo gravimetric analysis (DTA)

**Fig. 4:**
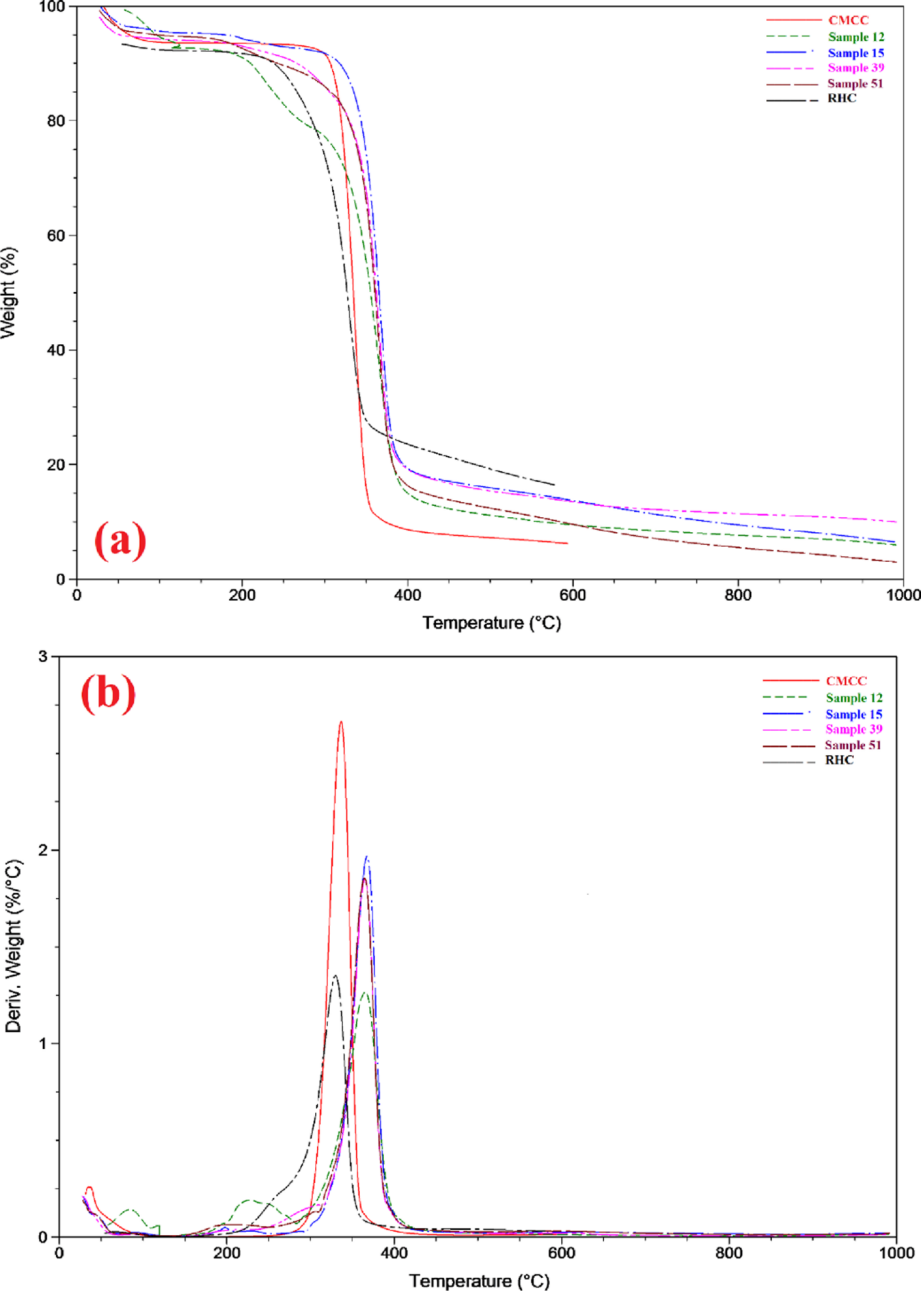
(**a**) TGA; (**b**) DTA analysis of CMCC, RHC, Samples 12, 15, 39, and 51.

TGA and DTA were measured to compare the thermal stability of extracted rice husk cellulose (RHC), synthesized cellulose acetate from CMCC, and extracted RHC at room temperature and 50 °C (samples 12, 15, 39, and 51, respectively). Figure 4 illustrates the TGA and DTA curves for extracted RHC and synthesized cellulose acetate assigned to the weight loss upon continuous heating to 1000 °C. The extracted rice husk showed three decomposition stages between 50 and 650 °C. The initial weight loss was approximately 100 °C due to water vaporization, and the moisture content was 10.63%. A sharper weight drop is observed at higher temperatures from 200 to 400 °C (85.53%). The DTA curve showed that the central peak associated with the thermal decomposition of crystalline cellulose was 353.20 °C, while the thermal decomposition of glycosidic links of cellulose and hemicellulose occurred at around 318.28 °C (7.42%). Finally, the residual mass (22.81%) attributed to lignin decomposition and silica occurred within a temperature range, starting well below 400 °C and persisting above 600 °C. TGA and DTA were also conducted for the four samples: 12, 15, 39, and 51. As shown in Fig. 4, the four samples exhibited a similar changing trend over the entire temperature range (50–1000 °C).

Meanwhile, the primary thermal decomposition occurred between 325.83 and 410.0 °C, and the peak in the DTA curves occurred around 364.0 °C. Thermal decomposition is characterized by extensive degradation of cellulose acetate, including depolymerization, dehydration, and cleavage of glucosyl links. The last step, which starts above 410.0 °C, represents the complete depolymerization of the degraded products, leaving a residue^23,33^.

## Conclusion

This study provides a simple approach for obtaining modified polymer materials from different natural sources using a new and very effective catalyst (Cu(ClO_4_)_2_.6H_2_O). Synthesizing di- and tri-cellulose acetate networks by controlling the reaction conditions is crucial in developing these materials. Cellulose di- and triacetates were obtained from cellulose extracted from rice husk and from commercial microcrystalline cellulose. The effects of temperature, catalyst weight, and time on the production of cellulose di- and triacetate were evaluated. The formation of tri-cellulose acetate increased with increasing time, temperature, and the weight of catalyst in all samples (Commercial microcrystalline and extracted rice husk cellulose) except commercial microcrystalline cellulose at 50 °C (samples 13–24) as explained before. The acetylation of the various celluloses under different conditions was confirmed by using FT-IR analysis. Compared with the synthesized cellulose acetate and extracted RHC, the thermal analysis showed that the synthesized cellulose acetate had better thermal stability than extracted RHC. While the X-ray diffraction indicated that the crystallinity of the synthesized cellulose acetate of commercial microcrystalline and extracted rice husk at room temperature and 50 °C (crystallinity of samples 12 (21.5%), 15 (23.6%), 39 (22.7%), and 51 (24.8), respectively) dramatically decreased compared to that of the extracted RHC (crystallinity = 30.2%).

## Electronic Supplementary Material

Below is the link to the electronic supplementary material.

Supplementary material 1

## Data Availability

The datasets used in this investigation are available for review upon request from the corresponding author of this paper.
