# Supplementary material for: Synthesis of di- and tri-cellulose acetate from rice husk cellulose and commercial microcrystalline by copper perchlorate catalyst
Source: Sci Rep. 2026 May 27;16:16422. doi: 10.1038/s41598-026-53816-6 (PMC13216330; doi:10.1038/s41598-026-53816-6)
Supplement: Supplementary file 1 — Supplementary material 1 [file 41598_2026_53816_MOESM1_ESM.docx]

**Supplementary materials**

**Synthesis of di- and tri-cellulose acetate from rice husk cellulose and commercial microcrystalline by Copper perchlorate catalyst**

Safaa Ragab, Amany El Sikaily, Ahmed El Nemr*

National Institute of Oceanography and Fisheries (NIOF), Kayet Bey, Elanfoushy, Alexandria, Egypt

*Corresponding Author E-mail: [ahmedmoustafaelnemr@yahoo.com](mailto:ahmedmoustafaelnemr@yahoo.com); [ahmed.m.elnemr@gmail.com](mailto:ahmed.m.elnemr@gmail.com)


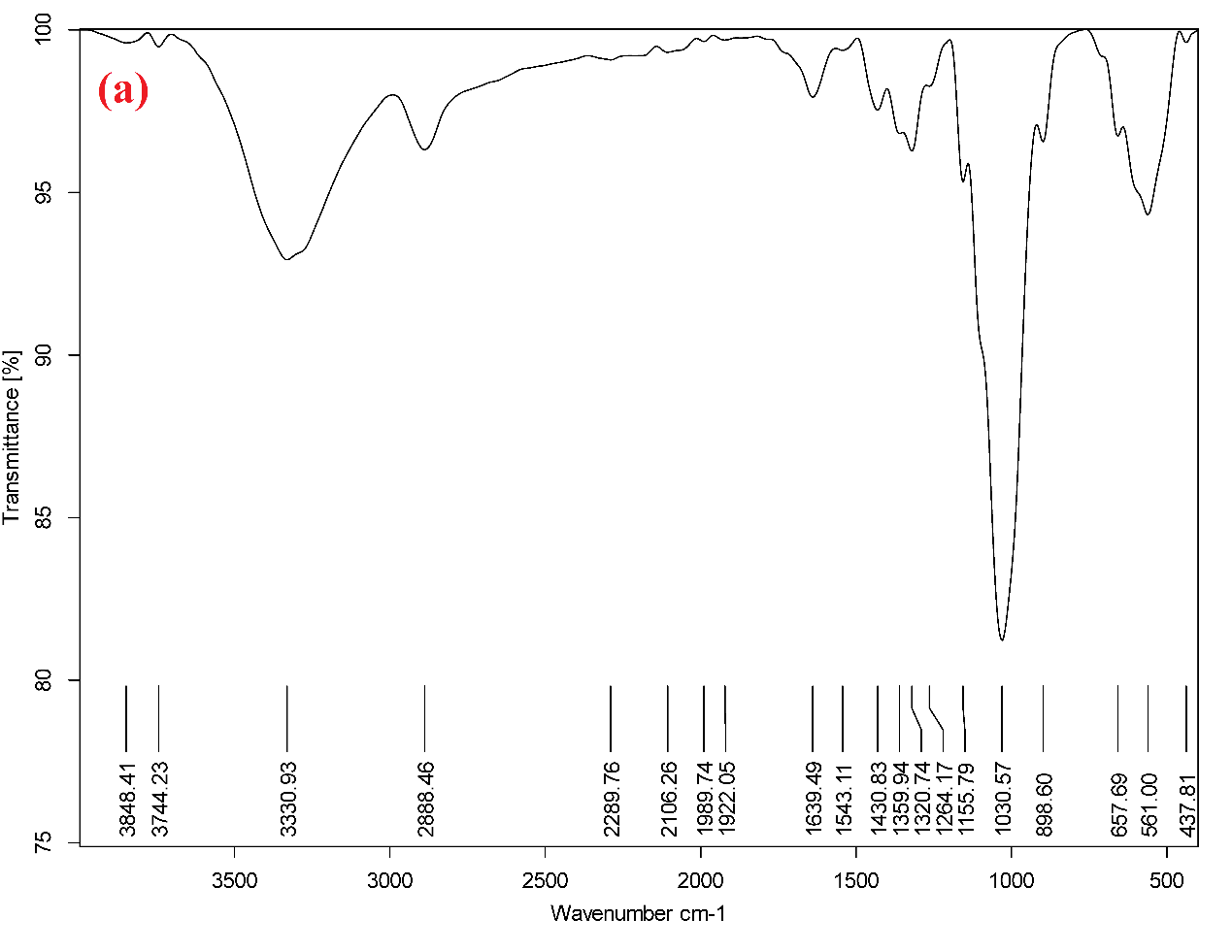


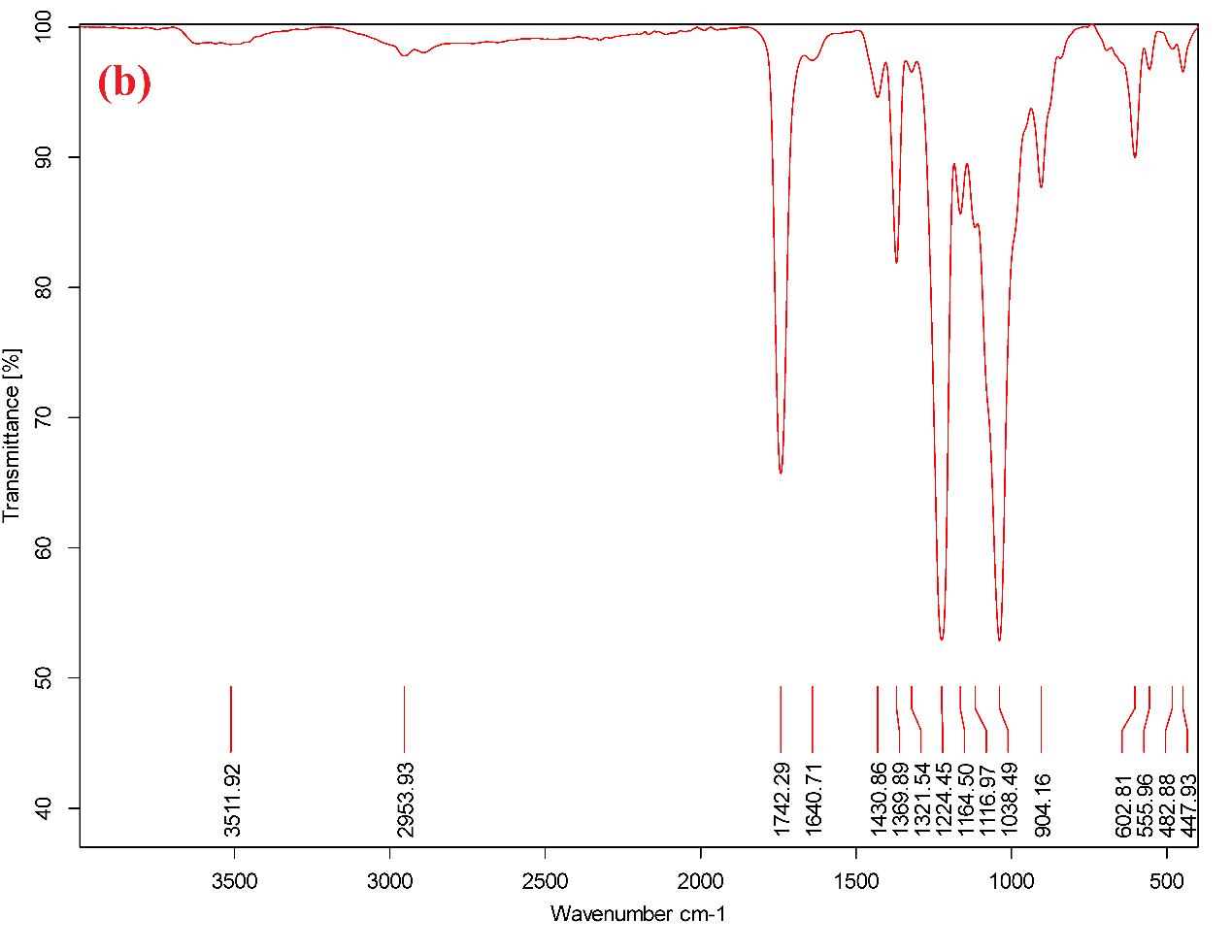


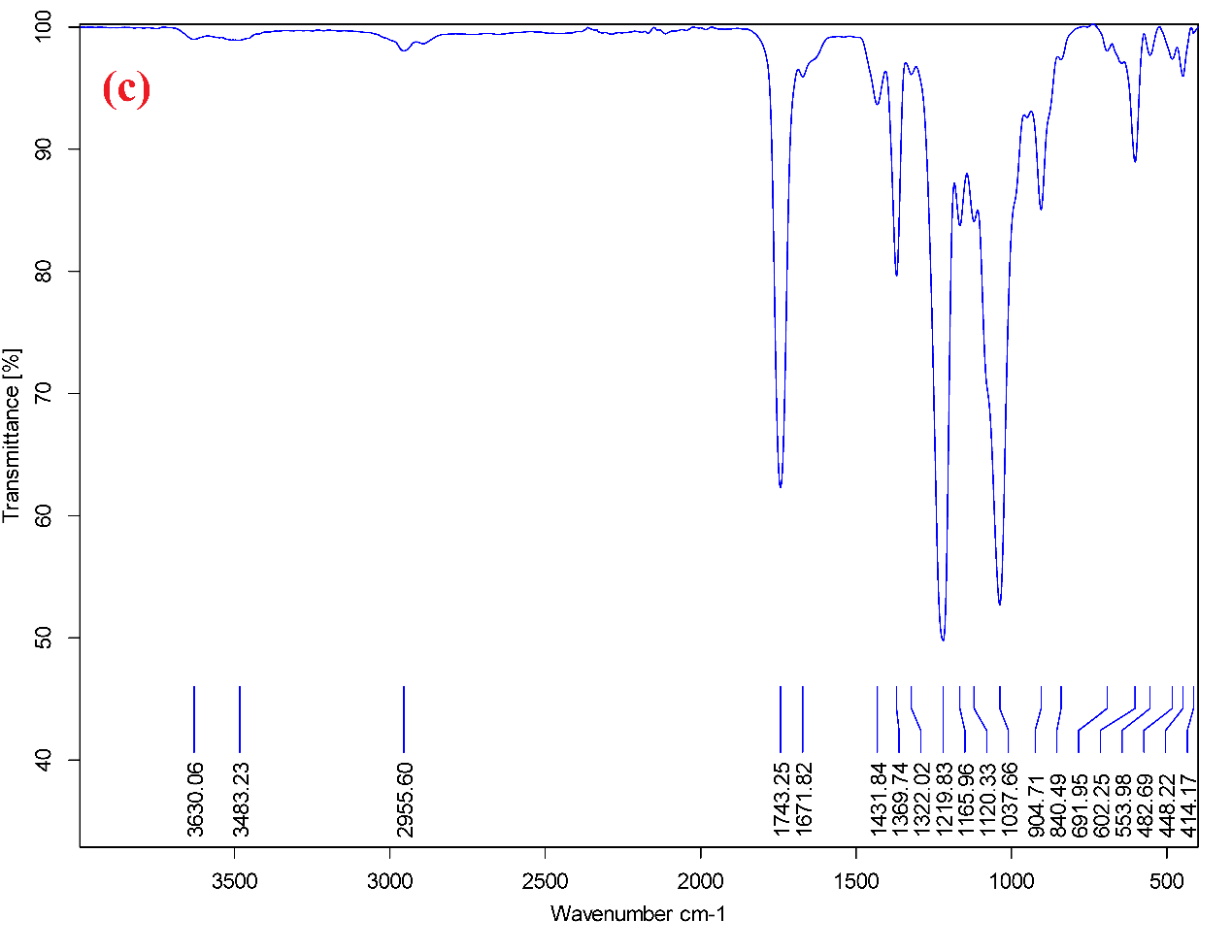


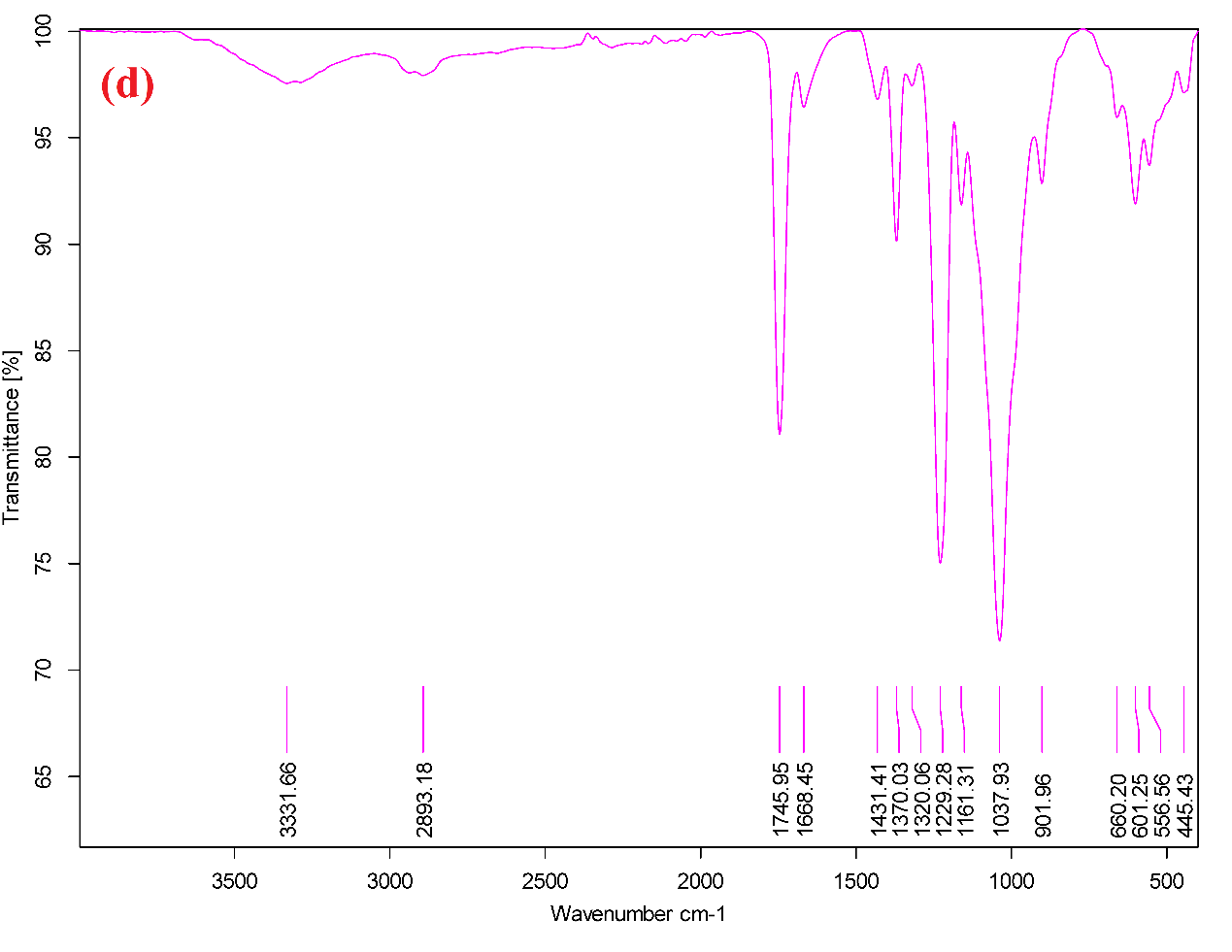


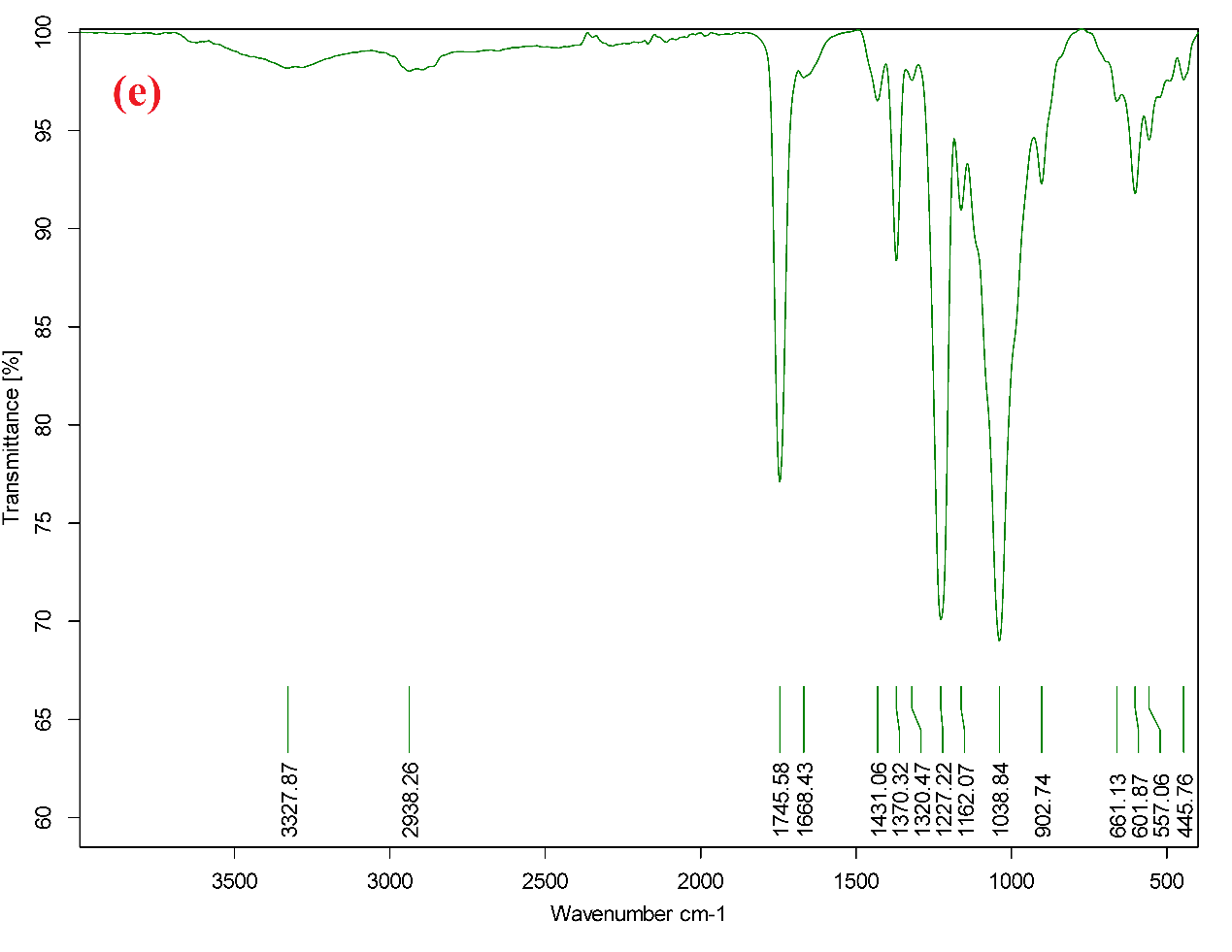


**Figure 1**. FTIR analysis of (a) rice husk microcrystalline cellulose, (b) Commercial microcrystalline cellulose acetate at room temperature (reaction 12), (c) Commercial microcrystalline cellulose acetate at (reaction 50 °C 15), (d) rice husk microcrystalline cellulose acetate at room temperature (reaction 39), and rice husk microcrystalline cellulose acetate at 50 °C (reaction 51).


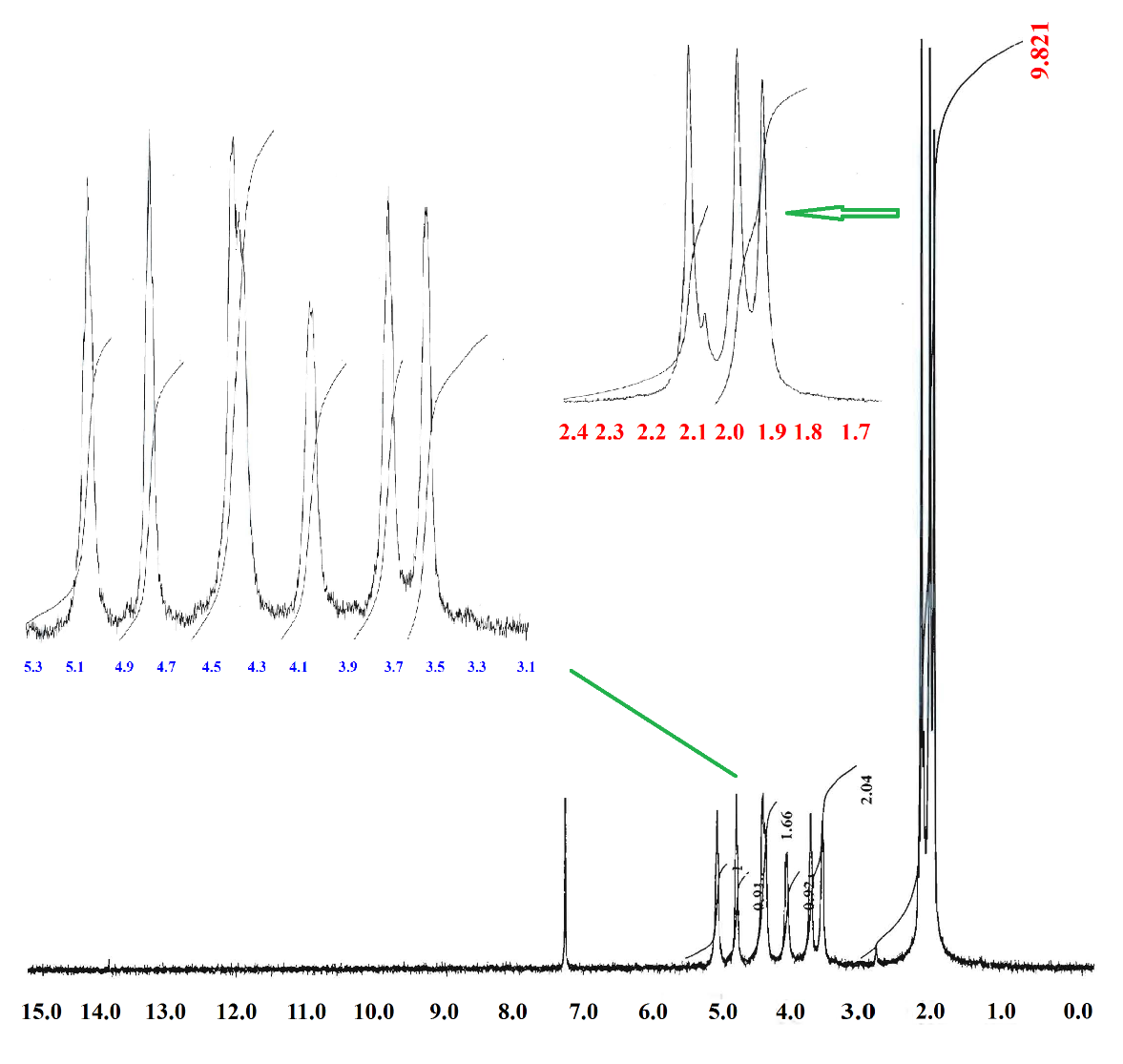


**Figure 2**. ^1^H-NMR analysis of Sample 12.


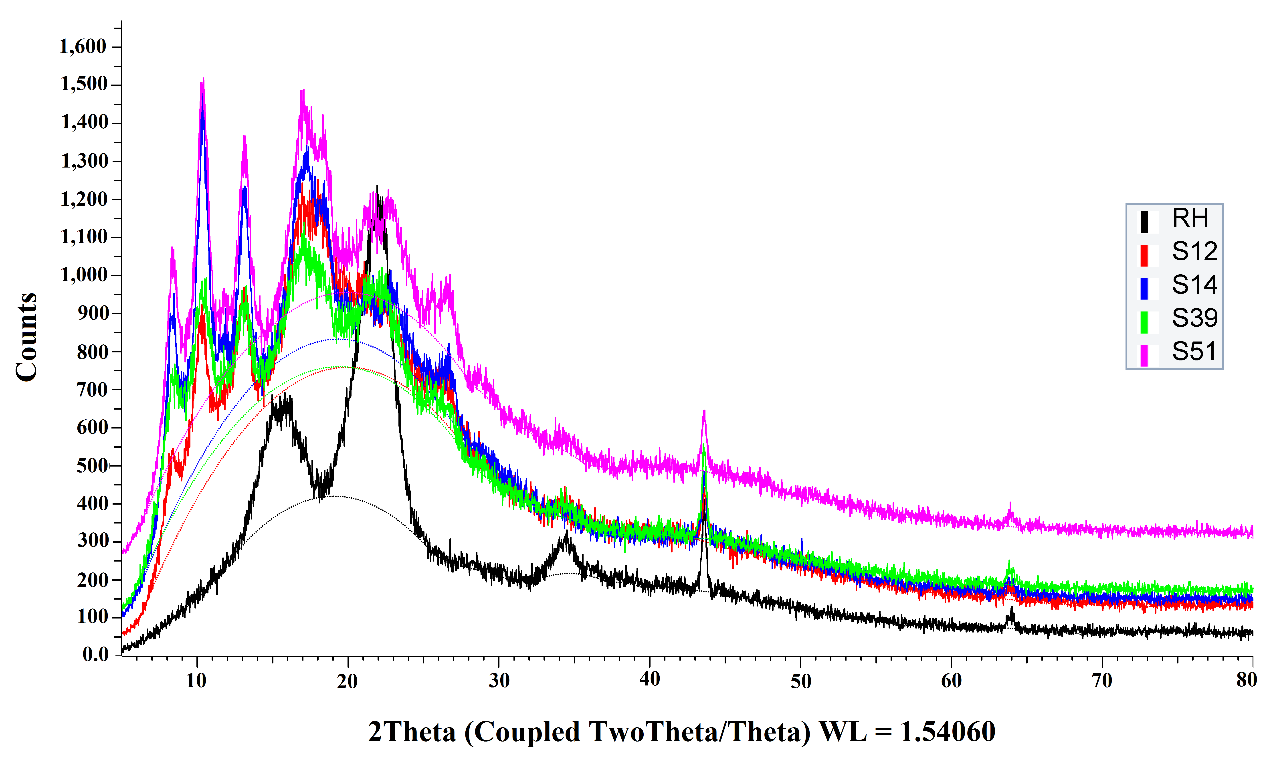


Fig. 3. The XRD profile of extracted rice husk cellulose.


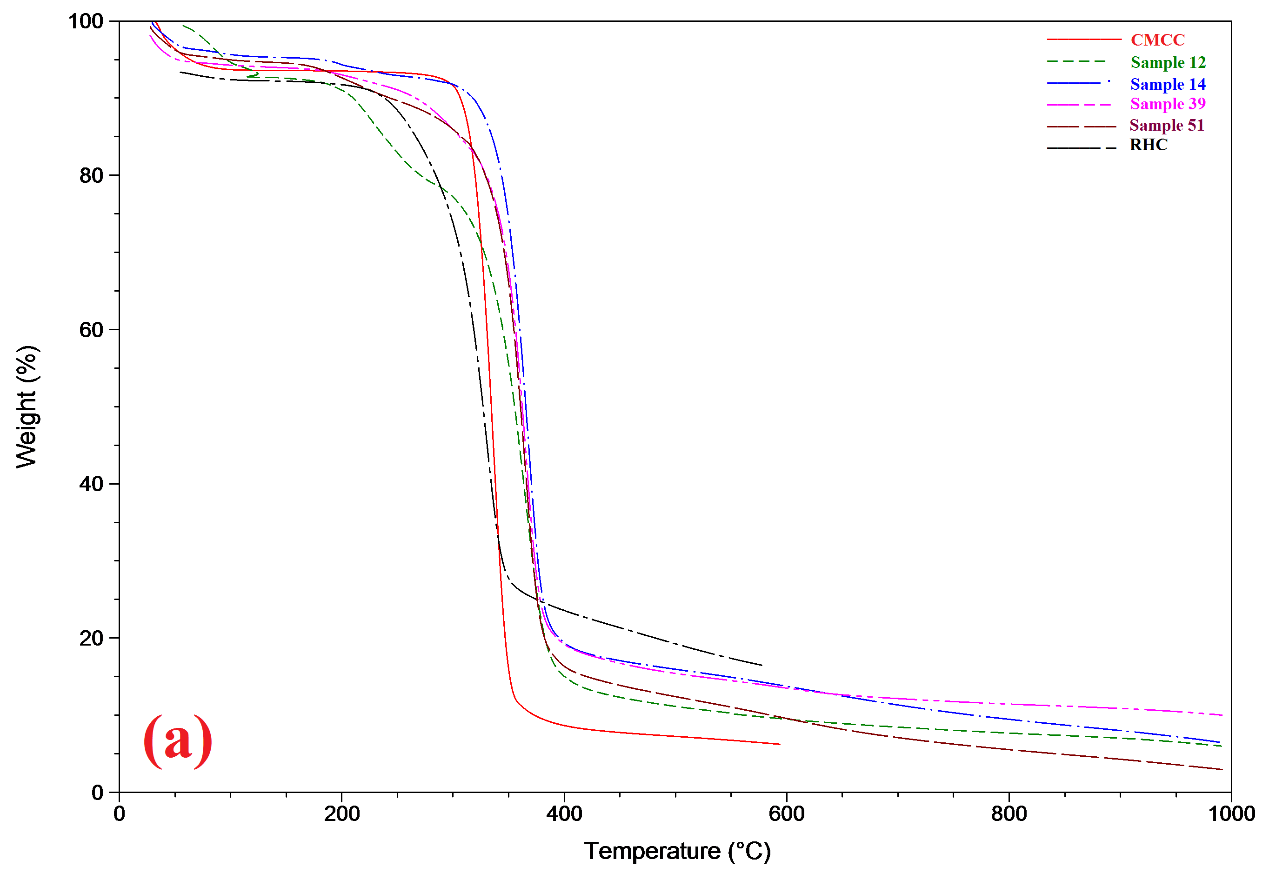


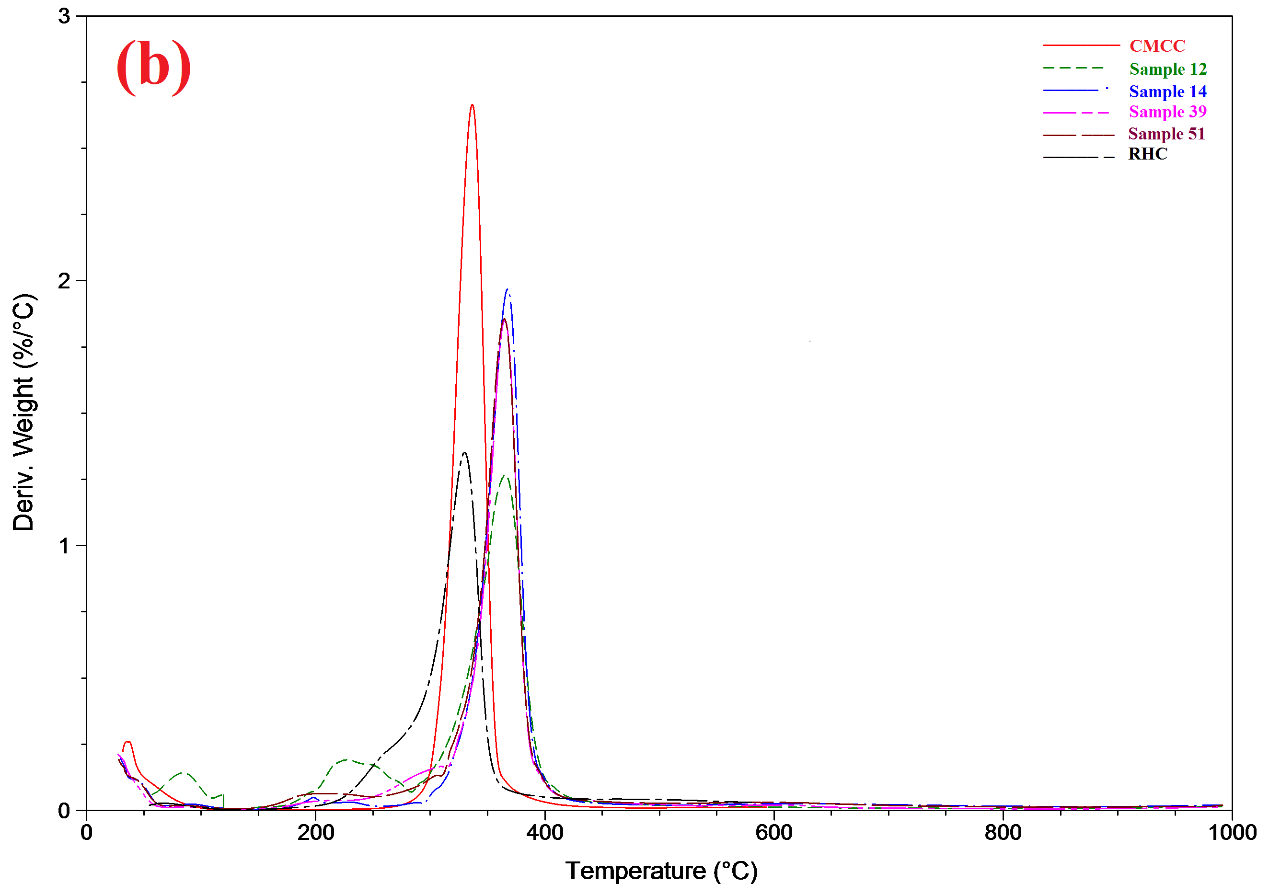


**Figure 4**. (a) TGA; (b) DTA analysis of CMCC, RHC, Samples 12, 14, 39, and 51.

*Degree of polymerization of cellulose acetate (DP)*

The degree of polymerization, weight average molecular weight (Mw), number average molecular weight (Mn), z average molecular weight (Mz), and polydispersity index (Mw to Mn (PDI)) of produced cellulose acetate were all assessed by GPC. Thus, molecular weight averages and the polydispersity index are highly helpful in determining the width of a polymer dispersion. Knowing a polymer-based product's molecular weight is crucial. A molecular weight distribution is shown by a bell curve, where the high end denotes a high molecular weight and the low end a low molecular weight. The sample is extremely polydisperse, comprising a range of molecules with different molecular weights, if a broad molecular weight distribution peak is seen. A monodisperse sample has primarily one tiny molecular range if its molecular weight distribution peak is sharp.

Table S1. GPC analysis of cellulose acetate Samples 12, 15, 39 and 51 prepared using different amount of Copper perchlorate hexahydrate catalyst in 15 mL acetic anhydride.

| Sample No. | M_W_ (g/mol) | Mn (g/mol) | M_Z_ (g/mol) | PDI | DP |
| --- | --- | --- | --- | --- | --- |
| 12 | 55634 | 14145 | 139634 | 4.720 | 208 |
| 15 | 50222 | 12997 | 132700 | 3.788 | 197 |
| 39 | 47310 | 15901 | 107021 | 4.165 | 178 |
| 51 | 41821 | 15165 | 101091 | 3.880 | 154 |

Mw: average molecular weight; Mn: Number average molecular weight; Mz: z average molecular weight; PDI: polydispersity index; DP: Polymerization degree. The DP calculated values were calculated and normalized as reported [1,2].

[1] El Nemr A, Eleryan A, Mashaly M, Khaled A (2021) Comparative study of synthesis of cellulose propionate from different sources using NIS as a New Catalyst. Polymer Bulletin. 78:4369–4386. <https://doi.org/10.1007/s00289-020-03313-1>

[2] El Nemr A, Eleryan A, Mashaly M, Khaled A (2021) Rapid synthesis of cellulose propionate and its conversion to cellulose nitrate propionate. Polymer Bulletin 78:4149–4182. <https://doi.org/10.1007/s00289-020-03317-x>
